# Traveling-Wave Convection with Periodic Source Defects in Binary Fluid Mixtures with Strong Soret Effect

**DOI:** 10.3390/e22030283

**Published:** 2020-02-29

**Authors:** Laiyun Zheng, Bingxin Zhao, Jianqing Yang, Zhenfu Tian, Ming Ye

**Affiliations:** 1 School of Mechanical Engineering, Ningxia University, Yinchuan 750021, China; zhenglaiyun@126.com; 2 School of Mathematics and Statistics, Ningxia University, Yinchuan 750021, China; y_jianqing@126.com; 3 Department of Aeronautics and Astronautics, Fudan University, Shanghai 200433, China; zftian@fudan.edu.cn; 4 Department of Earth, Ocean, and Atmospheric Science, Florida State University, Tallahassee, FL 32306, USA; mye@fsu.edu

**Keywords:** convection, instability, binary fluid mixtures, Rayleigh–Bénard, defect, traveling wave

## Abstract

This paper studied the Rayleigh–Bénard convection in binary fluid mixtures with a strong Soret effect (separation ratio ψ=−0.6) in a rectangular container heated uniformly from below. We used a high-accuracy compact finite difference method to solve the hydrodynamic equations used to describe the Rayleigh–Bénard convection. A stable traveling-wave convective state with periodic source defects (PSD-TW) is obtained and its properties are discussed in detail. Our numerical results show that the novel PSD-TW state is maintained by the Eckhaus instability and the difference between the creation and annihilation frequencies of convective rolls at the left and right boundaries of the container. In the range of Rayleigh number in which the PSD-TW state is stable, the period of defect occurrence increases first and then decreases with increasing Rayleigh number. At the upper bound of this range, the system transitions from PSD-TW state to another type of traveling-wave state with aperiodic and more dislocated defects. Moreover, we consider the problem with the Prandtl number Pr ranging from 0.1 to 20 and the Lewis number Le from 0.001 to 1, and discuss the stabilities of the PSD-TW states and present the results as phase diagrams.

## 1. Introduction

Thermal convection is a common phenomenon in nature, and has a wide range of engineering applications including nuclear systems, crystal growth and chemical vapor deposition, and industrial cooling. Thermal convection has been an active research topic for many years. This study concerns the Rayleigh–Bénard convection in binary fluid mixtures, of which complex time-dependence dynamic states appear in the vicinity of primary instabilities. The Rayleigh–Bénard convection is a paradigmatic system for the study of thermal convection problems (related to instabilities, bifurcations, self-organization and turbulence), and has been studied intensively since the 1980s [1]. The flow patterns in binary fluid mixtures are more interesting and complicated than those in one-component fluid because of the Soret coupling between the temperature and concentration fields. The Soret coupling reflects the influence of temperature gradients on concentrations of binary fluids, and its strength is characterized by the separation ratio ψ. The sign of ψ determines behaviors of binary mixtures. With ψ<0, destabilizing temperature gradients results in a competing concentration distribution, and its impacts on buoyancy force stabilize the flow field. For such a mixture of our interest, the primary bifurcation of the conduction state is a subcritical Hopf bifurcation that leads to rich dynamical behaviors near the onset of convection.

In recent decades, a substantial amount of effort has been spent to observe and reveal the dynamics of convection in binary fluid mixtures. Laboratory experiments have observed convective states taking the form of traveling waves (TW) [2], localized TW (LTW) [3,4], stationary overturning convection (SOC) [2], blinking [5,6], counterpropagating waves (CPW, or “chevrons”) [7,8], repeated transients [7,8] and dispersive chaos [9]. After these pioneering studies, many research by direct numerical simulations (DNS) were conducted to further understand and identify the mechanisms of the convective states, see e.g., References [10,11,12,13,14,15,16,17,18,19,20,21,22,23,24,25,26,27]. By DNS one can get easily the values at any point of the flow fields and extract from them information difficult or impossible to obtain in the experiments, this allows a better understanding of the convective states. However, simulations in three dimensions (3D) are prohibitively expensive at high Rayleigh numbers [1,28,29]. For low Rayleigh numbers, it is also challenging to investigate the problem by 3D simulations for a broad range of control parameters. Two-dimensional (2D) simulation, which is substantially less computational cost and found to capture many essential features of 3D convection [30,31], is a useful tool in fundamental research and has been widely used to study Rayleigh–Bénard convection, not only in binary fluids [10,11,12,13,14,15,16,17,18,19,20,21,22,23,24,25,26,27] but also in one-component fluid [28,29,32,33]. Barten and his coworkers [10] first reported the DNS results of convection in water-ethanol mixtures with separation ratio ψ=−0.6, and then studied in detail the TW and SOC states in binary mixtures with different ψ values in a narrow container of a single wavelength [11]. They analyzed the properties of the different fields, discussed the features of LTW state in extended containers with aspect ratio of Γ=20 and Γ=40 [12], here Γ is the ratio between length and height of the container, and also elucidated that the LTW states are stably and robustly sustained by strongly nonlinear mixing and complex flow-induced concentration redistribution [12,13]. Ning et al. [14] obtained a double localized traveling wave state in a large rectangular container of Γ=46. Batiste et al. obtained a novel state, viz. localized SOC (LSOC, or so-called “convectons") in the ${}^{3}$He-${}^{4}$He mixtures for the first time [34], and found that this state also exists in water-ethanol mixtures [15]. After that, multiple LSOC states such as anticonvectons, wall-attached convectons and stable traveling convectons, and collisions between different localized states were studied by different groups [16,17,18,19]. Recently, Smorodin et al. [20] discussed the influence of high-frequency transversal vibrations on the convective instability and pattern formation, and found that the unstable weakly nonlinear TW regime can be stabilized by means of high-frequency vibrations. We studied convection in binary mixtures with a weak separation ratio of ψ=−0.1, observed a undulation TW and five stable SOC states with different mean wavenumber $k=\frac{2\pi n}{\Gamma }$ (Here *n* is the number of roll pairs) in the container with the aspect ratio of Γ=12, and also discussed in detail the influence of fluid parameters and aspect ratio on the onset of convection and the properties of the five new stable SOC states [21]. Mercader and coworkers [22] analyzed the effect of a small inclination of angles α=0.01,0.03 and 0.05 on the 2D binary fluid convection with a negative separation ratio ψ=−0.1.

It was observed that in annular containers (with sufficiently large aspect ratio), solutions with slightly different wavelengths can fit into the container due to the Eckhaus instability, resulting in multiple TW state with different types of defects, such as drifting source-sink defects state and state with motionless source and sink defects [35,36,37,38,39]. Mercader et al. studied numerically the Eckhaus instability of traveling waves in large annular containers of aspect ratio around 80 for mixtures with three different separation ratios [23], and discussed the dynamics triggered by the Eckhaus instability for a mixture with separation ratio ψ=−0.127 [24]. In large rectangular containers with aspect ratio of Γ=40, transitions between different wavenumber traveling-wave patterns for separation ratio ψ=−0.25 were observed by Büchel and Lücke [25], and the dynamics was explained as a manifestation of Eckhaus instabilities. For this small separation ratio, Li and his coworkers [26] obtained an extended TW state with a periodic spatiotemporal dislocation defect. Ning et al. [27] studied the transitions from LTW to this state and finally to the TW state with increasing Rayleigh number. The aforementioned TW state with defects are mostly unstable, or pairs of rolls create and annihilate at a somewhat random location in the container. The purpose of this study is to use the high accuracy method [21] for studying the patterns arising in convection of binary fluid mixtures with strong separation ratio in a rectangle container of Γ=12. In such narrow rectangular container, the pattern selection dynamics is influenced by the presence of lateral boundaries and a stable TW state with periodic source defects (PSD-TW) is obtained in this study. In addition, the influences of the Prandtl and Lewis numbers on the stability of this state are discussed.

The rest of the paper is organized as follows. In Section 2 the mathematical model is presented along with a brief description of the numerical methods used for the calculations. In Section 3 we present the results and discussions, followed by the major conclusions of this study in Section 4.

## 2. Mathematical Physical Model

### 2.1. Governing Equations

**Figure 1:**
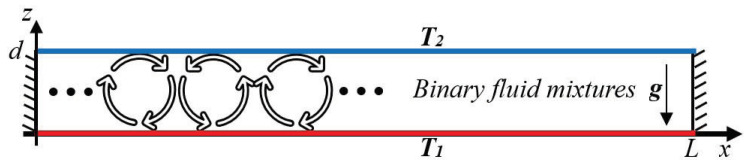
Sketch of the convection model.

We consider a Boussinesq binary fluid mixture confined in a 2D rectangular container of height *d* and length *L* with rigid boundaries. The container is heated uniformly from the bottom where the temperature is $T_{1}$, as shown in Figure 1.

The homogeneous gravitational field, $g=-ge_{z}$, is taken positive downward, and $e_{z}$ is the unit vector in the *z* direction. There is a positive temperature difference between the bottom and top boundaries $\Delta T=T_{1}-T_{2}$. The Rayleigh number is $Ra=\frac{\alpha gd^{3}\Delta T}{\kappa \nu }$, where α is thermal expansion coefficient, κ is thermal diffusivity and ν is kinematic viscosity. When the buoyancy term induced by the Rayleigh number exceeds a certain threshold value, convective motion starts. For pure fluids, the critical Rayleigh number for initial instability of Rayleigh–Bénard convection in an infinite layer is $Ra_{c}^{0}=1708$. For comparison we use the reduced Rayleigh number $r=Ra/Ra_{c}^{0}$ in discussion in Section 3.

Within the framework of the Boussinesq approximation, we use *d* as the length scale and the vertical thermal diffusion time $t_{d}=d^{2}/\kappa$ as the time scale. The velocity is scaled by κ/d. We further introduce the dimensionless temperature θ, concentration *c*, and pressure *p* as

$$\begin{array}{c} \theta =\frac{T-T_{0}}{\Delta T},\;\;\;c=\frac{\beta }{\alpha }\frac{\left(C-C_{0}\right)}{\Delta T},\;\;\;p=\frac{p^{*}d^{2}}{\rho_{0}\kappa^{2}}, \end{array}$$

and ignore the Dufour effect, where *T* is the temperature, *C* the concentration of the denser component, $T_{0}$ and $C_{0}$ denote the mean values along the height of the container in the reference state, i.e., the values at the mid-height of the container, $\rho_{0}$ is the fluid density at mean values $T_{0}$ and $C_{0}$, and β is the solutal expansion coefficient. With these, we obtain the dimensionless equations describing binary fluid convection:

(1) $$\begin{array}{c} \nabla \cdot v=0 \end{array}$$

(2) $$\begin{array}{c} v_{t}+\left(v\cdot \nabla \right)v=Pr\nabla^{2}v-\nabla p+RaPr\left[\left(1+\psi \right)\theta +\zeta \right]e_{z} \end{array}$$

(3) $$\begin{array}{c} \theta_{t}+\left(v\cdot \nabla \right)\theta =\nabla^{2}\theta \end{array}$$

(4) $$\begin{array}{c} \zeta_{t}+\left(v\cdot \nabla \right)\zeta =Le\nabla^{2}\zeta -\psi \nabla^{2}\theta \end{array}$$

where v=(u,w) represents the velocity field, ζ is defined as ζ=c−ψθ. In addition to the Rayleigh number Ra, the equations include three additional dimensionless parameters: the Prandtl number Pr, the Lewis number Le and the separation ratio ψ. They are defined as

$$Pr=\frac{\nu }{\kappa }\;\;,\;\;Le=\frac{D}{\kappa }\;\;,\;\;\psi =-\frac{\beta }{\alpha }\frac{\kappa_{T}}{T_{0}}=-\frac{\beta }{\alpha }S_{T}C_{0}\left(1-C_{0}\right).$$

Here $\kappa_{T}=C_{0}T_{0}\left(1-C_{0}\right)S_{T}$ is the thermodiffusion coefficient, $S_{T}$ the Soret coefficient and *D* the mass diffusivity coefficient of the denser component. For room temperatures ($10^{o}C$–$40^{o}C$) water-ethanol mixtures, Pr lines between 5 and 20, and Le around 0.01.

### 2.2. Boundary and Initial Conditions

Governing Equations (1)–(4) are subject to appropriate boundary conditions. We consider here all the boundaries to be no-slip and impermeable, with fixed temperatures at the top and bottom and no sideways heat flux. Thus, the boundary conditions are given

(5a) $$\begin{array}{cc} v=\frac{\partial \zeta }{\partial n}=0, & \;\mathrm{on}\;\partial \Omega \\ \theta =0.5, & \;\mathrm{at}\;z=0 \end{array}$$

(5b) $$\begin{array}{cc} \theta =-0.5, & \;\mathrm{at}\;z=1 \end{array}$$

(5c) $$\begin{array}{cc} \partial_{x}\theta =0, & \;\mathrm{at}\;x=0,\Gamma . \end{array}$$

where ∂Ω denotes the boundary of the container, and Γ=L/d is the aspect ratio. We consider in this study the initial condition takes the form of straight parallel rolls with basic wavenumber $k_{E}\approx \pi$ [11,20,23,24], as seen in many experiments, and set the initial rolls have a $O(10^{-6})$ quantum level amplitude envelopes which follow the shape of a Gaussian function with slightly asymmetrical peak at $x_{0}=\frac{2\Gamma }{3}$. Thus, the initial temperature field reads

(6) $$\theta \left(x,z;t=0\right)=10^{-6}\cos\left(\pi x\right)\exp\left(-\frac{{\left(x-x_{0}\right)}^{2}}{8}\right),$$

while the fields of velocity and concentration are zero.

### 2.3. Order Parameters

The heat transport by convection is measured by using the Nusselt number Nu, defined as the ratio of the total vertical heat flux through the fluid layer to that of the conductive solution [11,21],

(7) $$Nu=\frac{1}{\Gamma }\int_{0}^{\Gamma }\langle w\theta -\frac{\partial \theta }{\partial z}\rangle dx,$$

where angular brackets denote integration in the vertical direction, and $\int_{0}^{\Gamma }\langle w\theta -\frac{\partial \theta }{\partial z}\rangle dx$ is the total vertical heat flux through the whole fluid layer. Specific results of interest are $Nu_{c}$ and $Nu_{h}$ on the top and bottom walls, and the expression (7) reduce to $Nu_{c}=-\frac{1}{\Gamma }\int_{0}^{\Gamma }\frac{\partial \theta }{\partial z}{|}_{z=1}dx$ and $Nu_{h}=-\frac{1}{\Gamma }\int_{0}^{\Gamma }\frac{\partial \theta }{\partial z}{|}_{z=0}dx,$ respectively. For the conduction solution, the Nusselt Number is Nu=1. Thus, Nu−1 reflects the contribution of convection to heat flux transfer. The maximum vertical velocity ${\left|w\right|}_{max}$ is used to measure the magnitude of convective amplitude. In addition, since ${\left|w\right|}_{max}$ and Nu−1 cannot properly represent the concentration field of the nonlinear convective state, we use the mixing parameter [11] to characterize the magnitude of concentration variations. The mixing parameter *M* is defined by

$$M=\sqrt{\bar{\langle c^{2}\rangle }/\bar{c_{cond}^{2}}},$$

where the bars and brackets represent vertical and lateral averages, respectively. *M* is the variance of the concentration field reduced by its value in the conductive state. In a perfectly mixed mixture where all concentration deviations *c* from the mean vanish, *M* would be zero. On the other hand, in the conductive state, *M* equals to 1. For the unsteady flow, we mainly consider a quantity $\bar{F}$ over a period τ,

$$\bar{F}=\frac{1}{\tau }\int_{t}^{t+\tau }F\;dt,$$

where *F* represents any of the three variables, ${\left|w\right|}_{max},M$, and Nu.

### 2.4. Numerical Method

In this study, we use a compact finite difference (FD) method, being of fourth-order accuracy and high resolution, to solve numerically the governing Equations (1)–(4) with boundary conditions (5) in primitive variables. It is a projection method-based FD algorithm on staggered grids for the different fields and has been used successfully to in our previous works [21]. In the method, the fourth-order combined compact upwind (CCU45) scheme [40] with high resolution is used to approximate the nonlinear convective terms, the fourth-order symmetrical Padé compact scheme is used to discretize the viscous terms, and a fourth-order compact difference approximation is employed for approximating the first derivatives in the continuity equation. The pressure Poisson equation is approximated by a fourth-order compact difference scheme constructed on the nine-point 2D stencil [41] and calculated using a multigrid strategy to accelerate the iteration process.

The grid resolution for this calculation in 2D rectangular container with aspect ratio of Γ=12 are 241×21, and has been checked with calculation on a fine grids of 721×61 for accuracy. It shows that the maximum of differences in the order parameters (the Nusselt number Nu, convection amplitude ${\left|w\right|}_{max}$ and mixing parameter *M*) are less than 3% between the different resolutions for both high and low Prandtl numbers. In addition, the time step independence test has been conducted using multiple different time steps for the same case. It is found that the maximum of differences in the order parameters between the time step $4\times 10^{-5}$ and the very small time step $1\times 10^{-7}$ are less than 1%. To ensure firstly the accuracy and then to reduce the amount of calculation, the time step $4\times 10^{-5}$ is used for numerical simulation in this paper.

## 3. Results and Discussion

Below we describe our results for binary mixtures filling a rectangle container with aspect ratio of Γ=12. The Prandtl number Pr=10, the Lewis number Le=0.01, and separation ratio ψ=−0.6 were for water-ethanol mixtures [42] of mean ethanol concentration $C_{0}\approx 0.08$ and mean temperature $T_{0}=282$ K. These parameter values correspond to those used by Barten et al. [10] in their studies on fully developed traveling-wave convection. Then we discuss the effects of the Rayleigh number, Prandtl number and Lewis number on the stability of the PSD-TW state.

### 3.1. Spatiotemporal Evolution of the PSD-TW State

For the considered parameters Pr=10,Le=0.01, the primary bifurcation of conduction state takes place at a critical Rayleigh number $r_{c}=1.456$, and the final saturation state is a left-traveling LTW since computing from the asymmetric initial condition (6).

**Figure 2:**
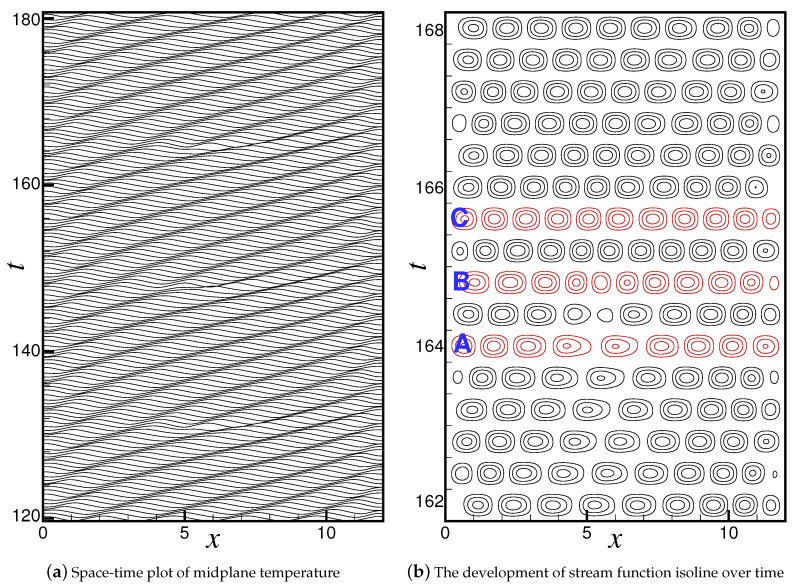
Flow field structure of traveling wave convection with source defects at r=1.8. “A”, “B” and “C” mark the cases shown in Figure 3.

Above $r_{c}$ and for r=1.8, with the rapid increase of convective amplitude, the right-traveling wave survives in the short time competing with the left-traveling wave, and the flow field eventually develops into a stable right-traveling TW state. Figure 2a shows the spatial-temporal evolution of the temperature field at r=1.8. In the figure, each curve represents the space-time plot of temperature in the midplane of the container at a given time. The time interval between two adjacent curves is $\Delta t=0.4t_{d}$, where $t_{d}$ is the vertical thermal diffusion time. The right-traveling TW state is not completely uniform in space and time. It is a spatial TW state with defects of period P=17.2. Traveling waves are generated at the left wall and annihilated after propagating to the right wall. In the process of wave propagation, the local wavelength is gradually elongated at a fixed location of x=5.0 in each period, and eventually divided into two parts. The TW state with this defect is usually referred to as a TW state with source defect. The spatial-temporal evolution of stream-function field is given in Figure 2b, which mainly shows the variation of the flow field when the source defect appears. We can see that one of the convective rolls is gradually stretched as it travels. At a certain time, the roll is split into two co-rotating rolls, and then a reverse rotating roll is generated between the two rolls. With the rapid increase of their convective amplitude, the new rolls grow to the same size as other rolls and propagate rightward along with other original rolls.

**Figure 3:**
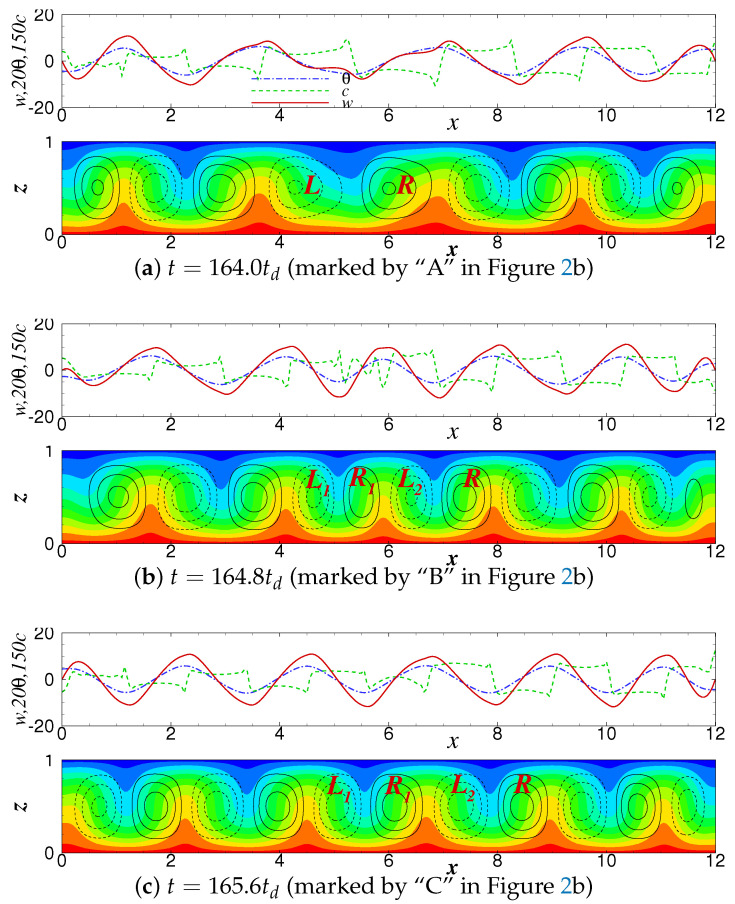
The flow fields and lateral profiles of the vertical velocity *w* (solid), temperature θ (dash dotted) and concentration *c* (dashed) in the vicinity of the time that defect occurs for r=1.8.

The flow fields and lateral profiles of the vertical velocity *w*, temperature θ and concentration *c* at the center line of the container at different time marked in Figure 2b are shown in Figure 3. Just before the occurrence of defect at $t=164.0t_{d}$, shown in Figure 3a, the local wavelength near x=5.0 is elongated enough and its shape is clearly different from those of other wavelengths. The roll marked by “*L*” is stretched obviously and the roll labeled by “*R*” is stretched too. As the defect occurs, it can be seen from Figure 3b that the wave is broken abruptly at x≈5.0 and a new wave crest is inserted between the two crests. The “*L*”-roll divides into two co-rotating rolls, i.e., “$L_{1}$” roll and “$L_{2}$” roll, and a reverse rotating “$R_{1}$” roll appears simultaneously between the two rolls, resulting in the previously stretched “R” roll is compressed back to its original size. The flow fields, especially the concentration field, change dramatically due to the change of wave phase by 180 degrees at x≈5.0.

Figure 3c shows that at time $t=165.6t_{d}$, the new roll has grown up, and its waveform is similar to that of the classic TW state. All of the rolls have almost the same shape and convective intensity. Particular attention is paid to the fact that the flow fields are not in the same phase. There is a phase difference between the velocity wave and the concentration wave. This leads to a concentration gradient between the adjacent rolls, and drives the rolls to propagate to the right. In addition, the lateral profile of the concentration is different from that of the velocity and temperature, showing a sharp-angle platform which is the typical feature of non-linear convection of traveling wave. It can be further seen that the shape of waves near the two lateral walls are slightly different from those inside the container, due to the influence of the lateral wall reflection.

### 3.2. Causes of Formation and Survival for the PSD-TW State

As shown in Figure 3a there are $n_{min}=4.5$ pairs of rolls in the system before the defect occurs. The corresponding mean wavenumber is ${\bar{k}}_{min}=\frac{2\pi n_{min}}{\Gamma }\approx 2.36$ and significantly smaller than the basic wavenumber $k_{E}\approx \pi$, which corresponds to the traveling wave of wavelength λ=2 consisting of 6 pairs of rolls [23,24,26,43]. The system is in the unstable regime of (k,r)-space, and the selection mechanism of an unstable pattern due to Eckhaus instability will trigger the processes of roll pair creation in the interior of the container [25]. When a new pair of rolls is generated, the number of convective rolls in the container is of $n_{max}=5.5$ pairs (see in Figure 3b) and the mean wavenumber is ${\bar{k}}_{max}\approx 2.88$ close to $k_{E}$, which brings the system returns to the stable wavenumber band. In general, the change in the number of roll pairs due to Eckhaus instability can generate a new stable globally uniform pattern with spatially uniform wavenumber inside the Eckhaus stable band [25,26,36,37]. However, for the parameters and boundary condition considered in this study, the convection state is different. One can observe from the figures that the annihilation of a convective roll at the right boundary takes a time interval of 1.2, while the generation of a roll at the left boundary requires a time interval of 1.5. The system will inevitably return to the state of $n_{min}=4.5$ roll pairs again since the annihilation frequency of the rolls is higher than the generation frequency. Then the new instability happens again. In addition, since the container considered here is a 2D rectangular one with two sidewalls, the flow structure is different from that in an annular system without sidewalls. Because of the confinement of the sidewalls, the traveling wave cannot form a TW state with uniform wavenumber after several oscillations. The system then oscillates back and forth between stable and unstable wavenumbers, and forms a PSD-TW state.

### 3.3. Variation of the Order Parameters

**Figure 4:**
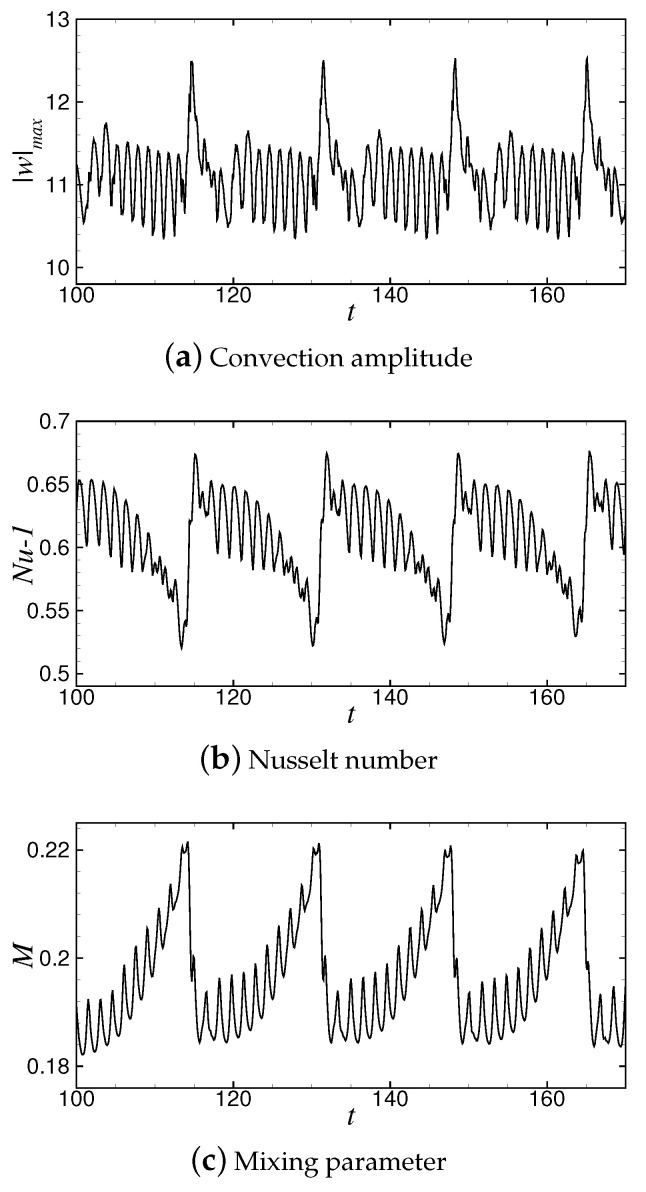
Variation of convection amplitude ${\left|w\right|}_{max}$, Nusselt number Nu−1 and mixing parameter *M* with time at r=1.8.

The variations of convective amplitude and Nusselt number with time at r=1.8 are shown in Figure 4a,b, respectively. It can be seen that the convective amplitude ${\left|w\right|}_{max}$ and the Nusselt number Nu−1 vary periodically. In one cycle, the intensity of ${\left|w\right|}_{max}$ and Nu−1 undergoes a process from strong to weak and to suddenly increase again.

Before a defect occurs, the convection intensity decreases with decreasing roll pairs in the container, and the values of ${\left|w\right|}_{max}$ and Nu−1 decrease to the minimum of 10.35 and 0.52, respectively. Thereafter, convection is strengthened instantaneously due to the generation of new convective rolls at the defect, and the convective amplitude ${\left|w\right|}_{max}$ reaches the maximum value of 12.50. Meanwhile, the convective heat transfer is sharply enhanced, and Nu−1 rapidly increases to the maximum value of 0.67. Next, with the adjustment of wavelength and wavenumber, the convective heat transfer decreases gradually, being accompanied by small oscillations which correspond to the generation and annihilation of convective rolls at the boundary.

Figure 4c shows the variation of the mixing parameter *M* with time at r=1.8. The trend of *M*-curve is obviously different from those of ${\left|w\right|}_{max}$ and Nu−1. With the increase of time, *M* increases to the maximum of 0.221 with 11 times oscillations, and then reduces rapidly to the minimum M≈0.182. Moreover, because the diffusion rate of concentration is lower than that of thermal diffusion, the change of *M* is slower than that of Nu−1, and the time taken for *M* to change from the maximum to the minimum is longer than that of Nu−1 and ${\left|w\right|}_{max}$. In fact, because the concentration diffusion velocity is smaller than the thermal diffusion velocity, the time when ${\left|w\right|}_{max}$ and Nu−1 reach the maximum in a period is not exactly the time when *M* reaches the maximum, and the difference between them is about $\Delta t\approx 0.7t_{d}$ vertical thermal diffusion time.

### 3.4. Influences of r,Le and Pr

**Figure 5:**
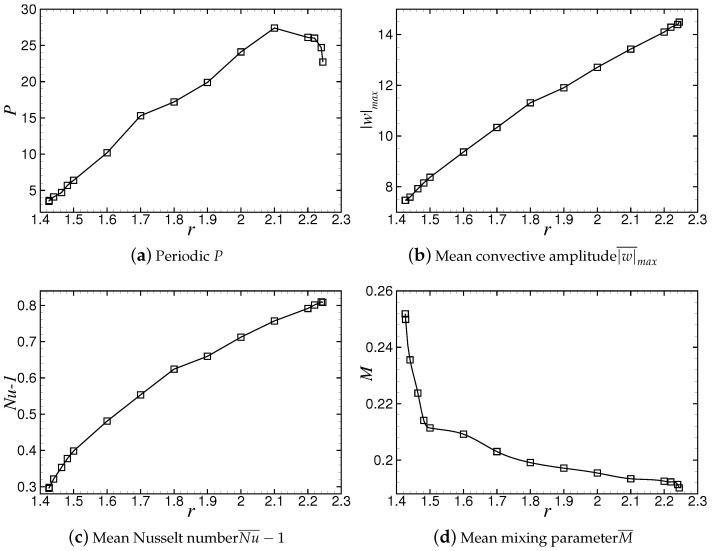
Variation of the period of defect occurrence and the order parameters with Rayleigh number *r*.

In the interval of 1.426≤r≤2.245, the PSD-TW state is stable, and the location of defects occurrence is x≈5.0. For r<1.426, the system undergoes a transition to a LTW state, and the transition is hysteretic. For r>2.245, the PSD-TW state becomes unstable, and the system eventually evolves into another type of TW state with aperiodic and more dislocated defects. This feature is different from that reported in References [26,27] where the TW state with a periodic spatiotemporal dislocation defect transitions to a TW state with uniform wavenumbers (without defects) with the increase of *r*. The variations of the period of defect *P* and order parameters ${\bar{\left|w\right|}}_{max}$, $\bar{Nu}-1$ and $\bar{M}$ with Rayleigh number *r* are plotted in Figure 5.

The period of defect occurrence *P*, as shown in Figure 5a, increases linearly with the increase of the Rayleigh number in the range of 1.426≤r≤2.100, while near the upper bound of the stable Rayleigh number range, 2.100≤r≤2.245, it decreases with increasing *r*. The P−value reaches a maximum 27.4 at r=2.100, which is approximately 8 times as large as the value at r=1.426. The mean convective amplitude ${\bar{\left|w\right|}}_{max}$ and Nusselt number $\bar{Nu}-1$ increase with Rayleigh number *r* in the stable range, as shown in Figure 5b,c. The trend of the mean mixing parameter $\bar{M}$ is opposite to that of the mean convective amplitude and Nusselt number, and decreases exponentially with increasing *r*, as shown in Figure 5d. In the stable range, the convective heat and mass transfer are enhanced with the increase of Rayleigh number, which leads to the fluid mixing becomes more uniform and the value of $\bar{M}$ decreases gradually. Near the lower bound of the stable range, 1.426≤r≤1.50, convection in the container is weak overall, any small increase in the Rayleigh number will have an obvious effect on the flow field, resulting a rapidly decrease of $\bar{M}$ with increasing *r*.

**Figure 6:**
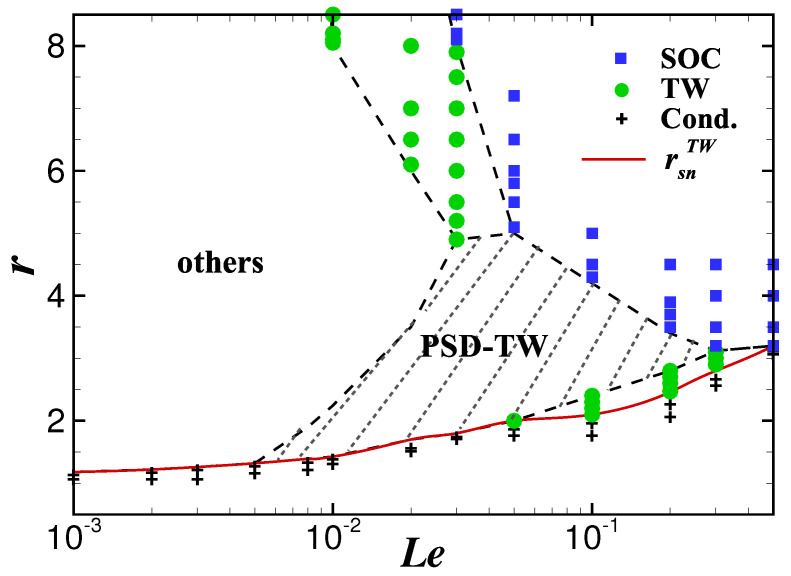
Le−r phase diagram for ψ=−0.60 and Pr=10. “Cond." represents the conduction state, and “others" refers to TW state with other types of defects.

To find the dependence of the stable PSD-TW state on the Lewis number, a large amount of numerical simulations were carried out in a wide range of 0.001≤Le≤1 for the fixed Prandtl number Pr=10. Figure 6 presents phase diagram of the Rayleigh number *r* versus the Lewis number Le.

The PSD-TW state is stable inside the shaded area in Figure 6, corresponding to the Lewis number ranging from 0.005 to 0.3. In this interval, the stable range first widens and then narrows with the increase of the Lewis number, and is the widest near Le=0.05. We note that at the upper edge of the shaded area, for different intervals of the Lewis number, a PSD-TW state transitions to a TW state with aperiodic and/or dislocated defects (0.005≤Le<0.03), or a TW with uniform wavenumbers (0.03≤Le<0.05), or a SOC state (Le≥0.05). For the first two cases, the stability changes again with further increasing *r*, and the system eventually transitions to a SOC state at a sufficiently large Rayleigh number. The bottom red curve corresponds to the saddle-node bifurcation points $r_{sn}^{TW}$, under which the system undergoes a transition to the conductive state and the finite amplitude convection state abruptly disappears. Starting from the saddle-node points and gradually increasing *r*, we find that the TW state appears earlier than the PSD-TW state in the range of 0.05<Le<0.3, while in the range of 0.03<Le<0.05 it is opposite.

**Figure 7:**
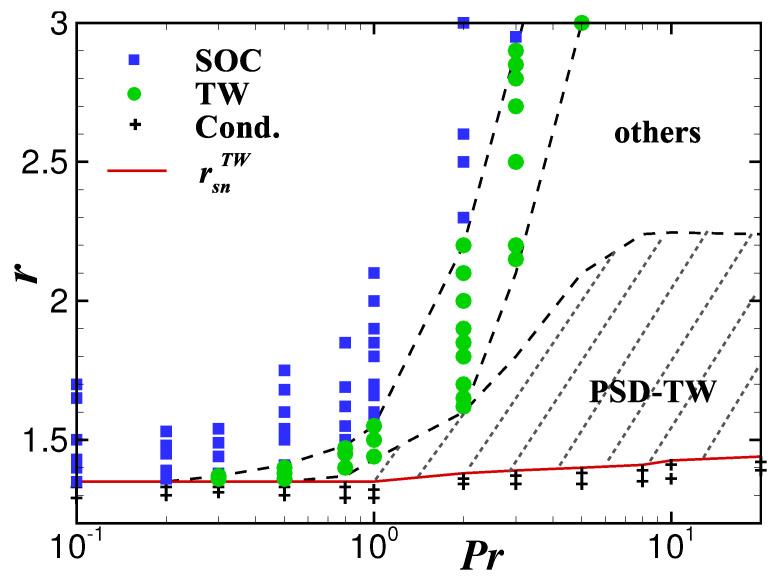
Pr−r phase diagram for ψ=−0.60 and Le=0.01.

The influence of Pr on the PSD-TW state is shown in Figure 7 while we keep Le=0.01 constant.

In the shaded area of the Pr−r phase diagram the PSD-TW state is stable and the range of the Prandtl number corresponding to this area is from 0.8 to 20. The existence range of stabe PSD-TW widens with increasing Pr and its width does almost not change while Pr≥10. Please note that in the small Prandtl number regime, there is no stable PSD-TW state, and in the very small Prandtl number regime, there is not even any form of TW state. Starting from the saddle-node bifurcation point $r_{sn}^{TW}$ and increasing with *r*, the process from a TW-type state to the final SOC state is different depending on the interval of the Prandtl number. In the interval of 2≤Pr≤20, the system undergoes successively a stable PSD-TW, TW state with aperiodic and/or dislocated defects and TW with uniform wavenumbers, while in the interval of 2≤Pr≤20 it only experiences the TW with uniform wavenumbers.

## 4. Conclusions

In this paper, by using the high-order compact FD algorithm to numerically solve the fully coupled hydrodynamic field equations, we studied a traveling wave state with source defects of convection in binary fluid mixtures with a negative separation ratio. The selected physical parameters (ψ=−0.6,Pr=10, and Le=0.01) of the mixtures is representative of water-ethanol mixtures at room temperature. For the considered parameters, we found that the new PSD-TW state is stable when the reduced Rayleigh number *r* ranges from 1.426 to 2.245, and the defect always occurs at about x=5.0, regardless of the Rayleigh number. The results show that besides the physical parameters, the pattern formation in the Rayleigh–Bénard convection depends on many factors, such as initial conditions and aspect ratio. We believe that in other containers with different aspect ratios, the location of defects may no longer be x=5.0. The state transitions to a TW state with aperiodic and more dislocated defects at r=2.246 and then to a TW state with uniform wavenumber. The period of defect occurrence increases linearly with the increase of Rayleigh number when r<2.100, and then decreases rapidly for *r* closing to the upper bound of the stable range. It is also found that the combined action of the Eckhaus instability and the difference between the creation and annihilation frequencies of convective rolls at the left and right walls is the reason for the formation and maintenance of the PSD-TW state. In addition, the variation of the order parameters with time was discussed. In a cycle, ${\left|w\right|}_{max}$ and Nu−1 first decrease with increasing time, after reaching their local minima before the occurrence of a defect, they increase rapidly with increasing time.

Our calculations at ψ=−0.6 done for 0.001≤Le≤1 and 0.1≤Pr≤20 showed that the PSD-TW state is stable for the Lewis number ranging from 0.005 to 0.3 or the Prandtl number ranging from 0.8 to 20. Upon decreasing Pr or increasing Le beyond the reference value Pr=10 and Le=0.05, the existence range of stable PSD-TW state on the upper branch narrows quickly. On the other hand, decreasing Le eventually pinches off their existence range, while increasing Pr hardly changes their existence range.
